# Kienbock's disease: Case report and review of the literature

**DOI:** 10.1016/j.radcr.2025.06.066

**Published:** 2025-07-19

**Authors:** Soukaina Beyyato, Hajar Slaoui, Hajar Ouazzani, Ismail Chaouche, Amal Akammar, Nizar El Bouardi, Meriem Haloua, Moulay Youssef Alaoui Lamrani, Meryem Boubbou, Mustapha Maaroufi, Badreeddine Alami

**Affiliations:** a Department of Radiology and Interventional Imaging, CHU Hassan II, FEZ, Sidi Mohammed Ben Abdellah University, Fes, Morocco; b Department of Radiology Mother and Child and Interventional Imaging, CHU Hassan II, FEZ, Sidi Mohammed Ben Abdellah University, Fes, Morocco

**Keywords:** Kienböck’s disease, Lunate bone, Osteonecrosis, Wrist pain, Magnetic resonance imaging

## Abstract

Kienböck’s disease is a rare form of avascular necrosis affecting the lunate bone, typically seen in young adults and often resulting in chronic wrist pain and functional impairment. We present a case-based review of 3 young adults (2 males aged 20 and 30 years, and 1 female aged 28) with progressive dorsal wrist pain, reduced grip strength, and limited range of motion. Imaging revealed advanced lunate osteonecrosis, with Lichtman staging ranging from stage IIIa to IV. MRI and CT played a central role in diagnosis and treatment planning. Management varied according to disease stage, from conservative immobilization to surgical intervention. This report highlights the importance of early diagnosis, accurate staging, and tailored treatment strategies based on clinical presentation and surgical expertise.

## Introduction

Kienböck’s disease, also known as avascular necrosis of the lunate, was first described by Robert Kienböck in 1910 [1]. It is a rare condition that results from impaired blood supply to the lunate, leading to bone necrosis, structural collapse, and secondary degeneration of the carpal joints. Although the exact cause remains unclear, proposed contributing factors include repetitive microtrauma, anatomical variations such as negative ulnar variance, venous congestion, and systemic conditions like lupus or corticosteroid use [2,4].

The disease most commonly affects young adults between the ages of 20 and 40 and shows a male predominance [5]. Clinical presentation is often nonspecific, including dorsal wrist pain, reduced grip strength, and limited range of motion, which may delay diagnosis. Imaging, particularly MRI, plays a central role in identifying early ischemic changes before radiographic abnormalities appear [2,6].

The Lichtman classification system is widely used to stage the disease, ranging from normal radiographs (stage I) to pancarpal arthritis (stage IV) [3]. However, this classification does not always correlate with clinical symptoms or predict the optimal treatment, which remains controversial and varies depending on surgeon experience [7,8].

In this article, we present 3 illustrative cases of Kienböck’s disease, emphasizing clinical presentation, imaging findings, disease staging, and treatment outcomes, followed by a discussion integrating current literature.

## Case presentation

Case 1: A 20-year-old right-handed male with no significant personal or family medical history presented with a 4-month history of progressive right wrist pain. The pain was mechanical, exacerbated by wrist dorsiflexion and gripping, and unresponsive to over-the-counter analgesics. He denied any history of trauma, systemic disease, or steroid use.

On physical examination, tenderness was noted over the dorsal aspect of the wrist, particularly at the lunate fossa. Active dorsiflexion and palmar flexion were both reduced (30° and 40° respectively), with decreased grip strength compared to the contralateral side. No edema or erythema was present. Laboratory tests including inflammatory markers (CRP < 3 mg/L, ESR 5 mm/h) were within normal limits.

**Fig. 1:**
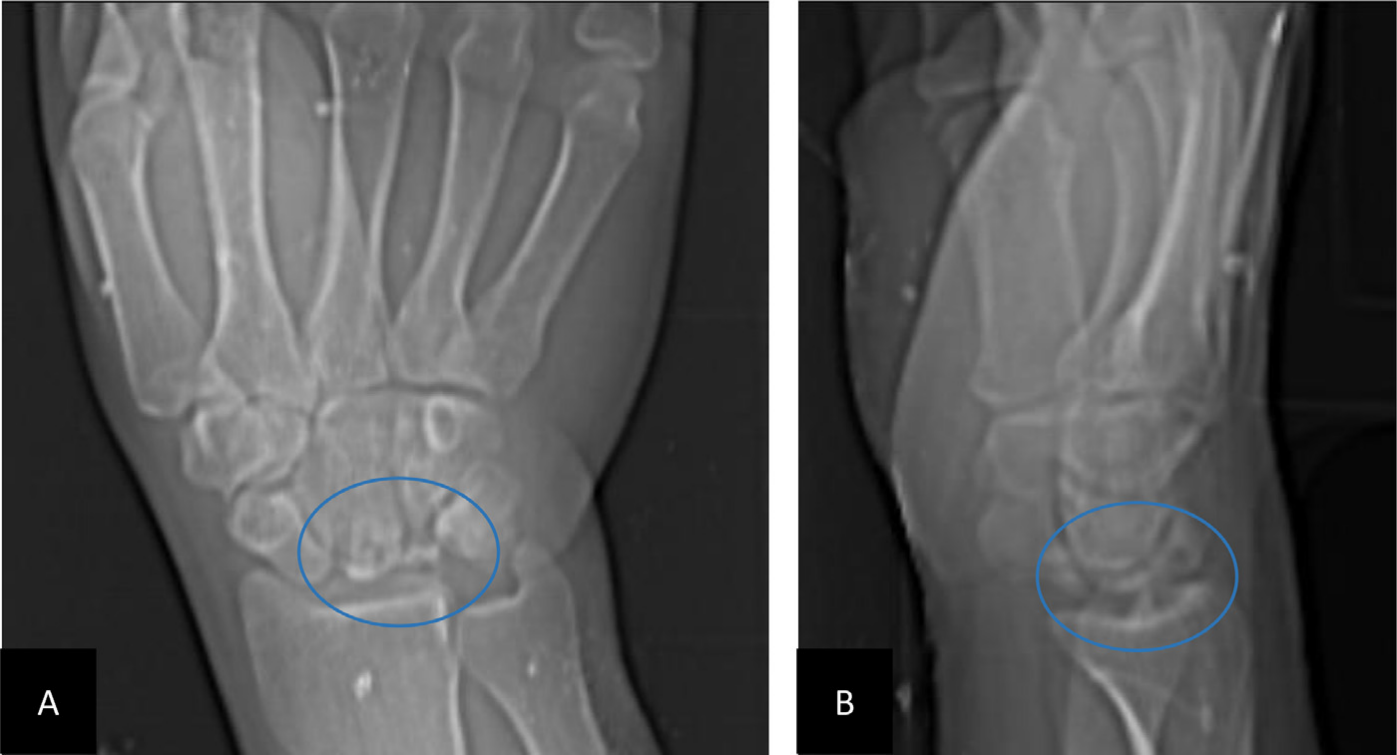
X-ray of the right wrist: the anteroposterior (A) and lateral (B) views revealed a collapsed and heterogeneous lunate with no involvement of the other carpal bones.

**Fig. 2:**
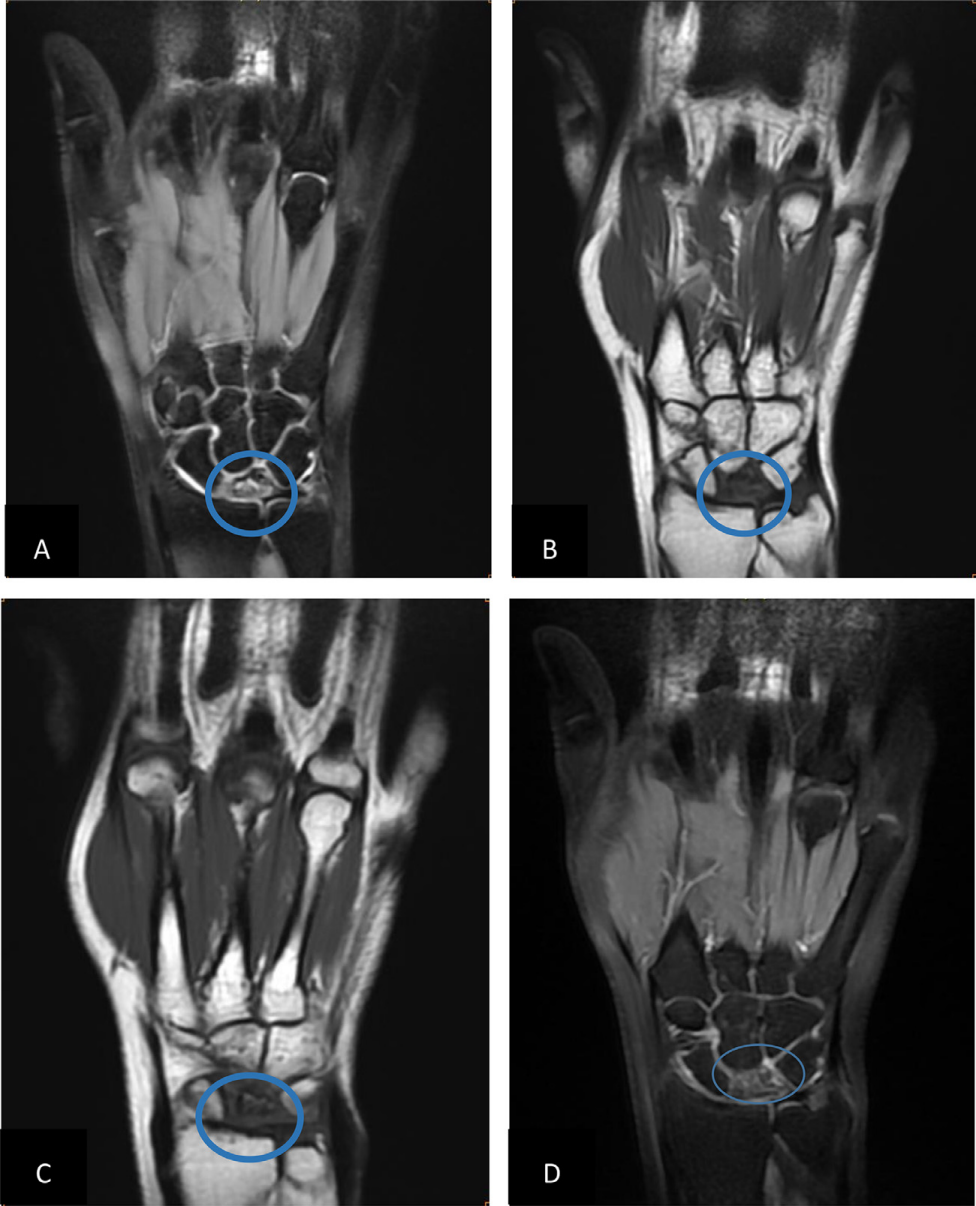
MRI of the right hand with coronal PD Fat Sat sequence (A), T1-weighted coronal sequence (B, C), and T1-weighted coronal sequence with fat suppression and gadolinium injection (D) revealed a collapsed lunate with homogeneous T1hyposignal and heterogeneous DP hypersignal, predominant in its anterior horn, slightly enhanced after contrast.

Standard wrist radiographs revealed collapse and fragmentation of the lunate with preserved joint spaces (Fig. 1). MRI demonstrated heterogeneous low T1 signal intensity with diffuse bone marrow edema. Postcontrast sequences showed reduced enhancement of the lunate, consistent with stage IIIa Kienböck’s disease (Fig. 2). Conservative management with wrist immobilization in a neutral position and activity modification was initiated, and the patient was referred to orthopedic surgery for further evaluation.

Case 2: A 30-year-old left-handed male presented with a 6-month history of gradually worsening left wrist pain and weakness. He reported difficulty performing manual tasks at work. There was no known trauma or medical history.

Clinical examination revealed tenderness over the dorsal radiolunate area, mild edema, and limited wrist dorsiflexion (20°) and radial deviation. Grip strength was markedly decreased. Laboratory tests including calcium, phosphorus, and inflammatory markers were normal.

**Fig. 3:**
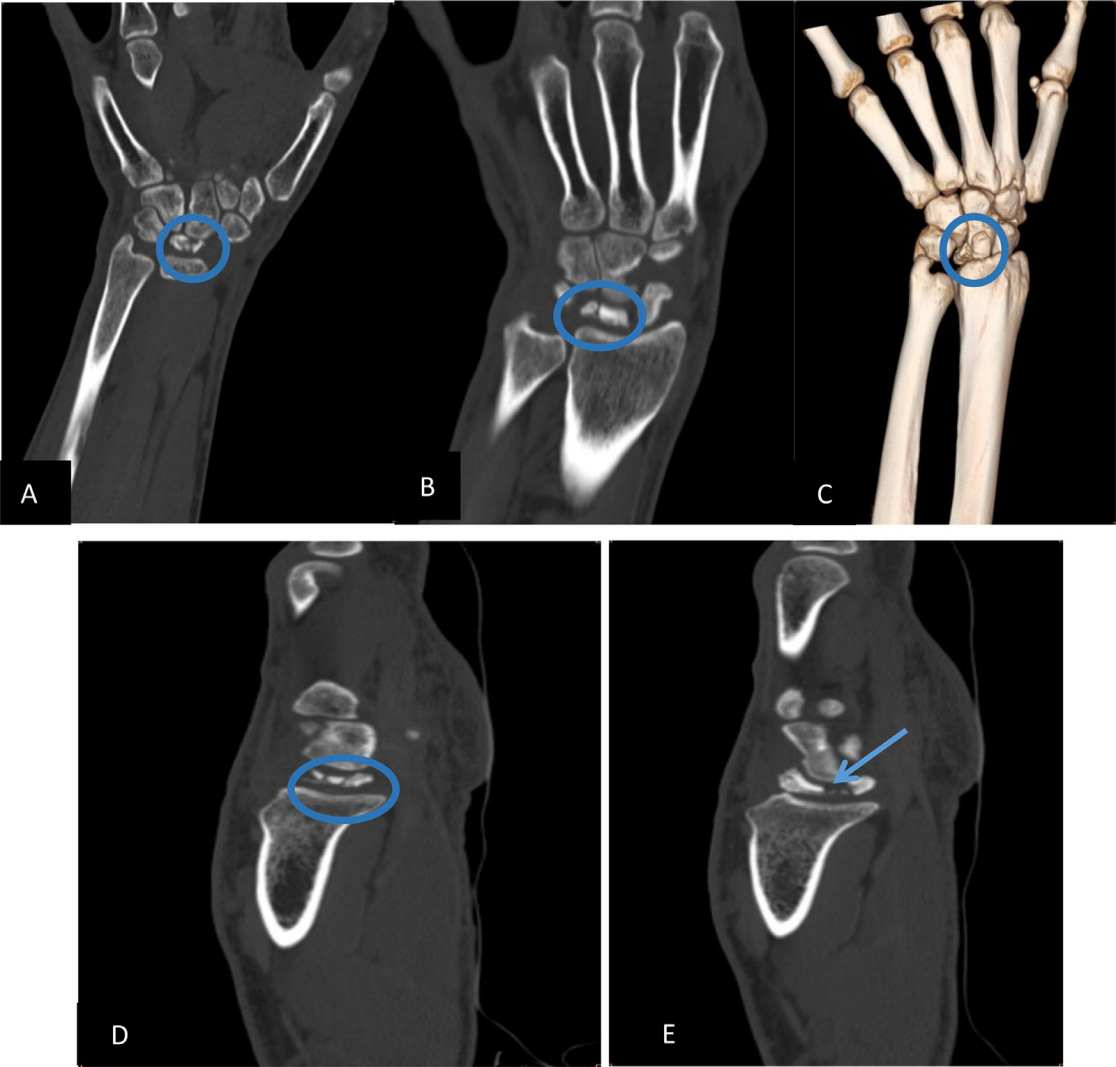
CT of the left wrist in coronal (A and B), sagittal (D and E), and 3D (C) sections revealing sclerosis and fragmentation of the lunate with articular erosion.

CT imaging showed lunate fragmentation, sclerosis, and articular erosion at the radiolunate joint (Fig. 3). MRI was not performed due to equipment unavailability. Based on radiographic and CT findings, stage IV Kienböck’s disease was diagnosed. The patient underwent proximal row carpectomy followed by a structured rehabilitation program. At 3-month follow-up, he reported improved pain and functional range of motion.

Case 3: A 28-year-old female presented with a 3-month history of right wrist pain, aggravated by activity and resistant to NSAIDs. She had no significant past medical or family history. She also noted increasing difficulty with tasks requiring grip and extension.

Examination revealed tenderness over the lunate, reduced dorsiflexion (25°), palmar flexion (35°), and grip weakness. There was no swelling or skin discoloration. Laboratory studies were normal.

**Fig. 4:**
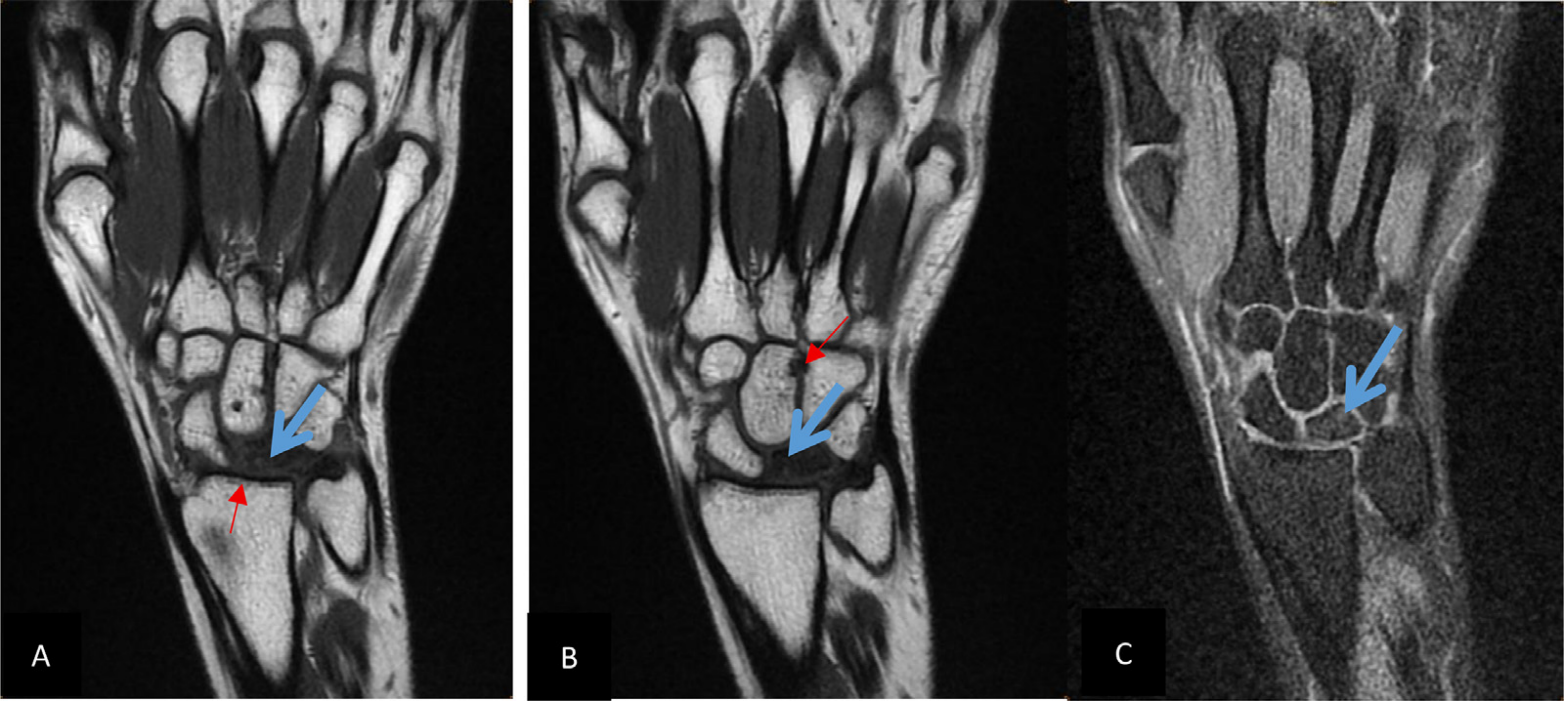
MRI of the right hand T1-weighted coronal sequence (A and B), and T1-weighted coronal sequence with fat suppression and gadolinium injection (C) demonstrates a hypointensity of the lunate bone and collapse (blue arrow) with osteoarthritic changes in radio lunate and mid-carpal joints (red arrow).

MRI demonstrated a flattened and fragmented lunate with low signal intensity on T1, and multiple subchondral cysts. Postcontrast images revealed global nonenhancement of the lunate. Pancarpal degenerative changes were also observed (Fig. 4). The diagnosis of stage IV Kienböck’s disease was established. The patient underwent total wrist arthrodesis. Postoperative recovery was uneventful, and she regained basic daily function with minimal discomfort.

## Discussion

Kienböck’s disease is characterized by progressive osteonecrosis of the lunate due to vascular compromise. It typically affects young adults, with a male predominance and unilateral presentation [2,4]. In our cases, all patients were in this demographic and presented with typical symptoms: dorsal wrist pain, grip weakness, and restricted motion.

The pathophysiology is multifactorial, involving repetitive mechanical stress, anatomical predisposition (e.g., negative ulnar variance), and occasionally systemic factors such as autoimmune disease or steroid exposure [4,7]. None of our patients had systemic risk factors.

Symptoms typically include dorsal wrist pain, reduced grip strength, and limitations in motion, especially in dorsiflexion, as observed in our patients. Tenderness on palpation of the lunate is common, while swelling may be minimal or absent.

Standard radiographs can detect changes like lunate sclerosis, collapse, and fragmentation. CT is more precise in evaluating bone architecture and articular surface integrity [8]. MRI is essential for early diagnosis, assessing vascularity, and guiding prognosis [5,9].MRI typically reveals decreased T1 signal intensity, with T2 signal ranging from hyperintense in early stages to hypointense in chronic sclerosis. In our cases, MRI confirmed diagnosis and guided staging in 2 patients; CT alone was sufficient in 1 advanced case.

The Lichtman classification remains the standard for radiologic staging, though it does not always reflect pain severity or functional limitation [3,7] (Table 1).

**Table 1:** Lichtman classification along with corresponding imaging findings and treatment approaches.

Disease stage	Radiographic findings	MRI findings	Treatment
I	Normal	T1 hypointense, T2 variable	Immobilization
II	Lunate sclerosis	T1 hypointense, T2 variable	Joint leveling (ulnar shortening)
IIIA	Lunate collapse, preserved carpal alignment	T1 hypointense, T2 edema	Revascularization, osteotomy
IIIB	Lunate collapse + fixed scaphoid rotation	T1 hypointense, T2 hypointense	Limited intercarpal fusion, PRC
IV	Pancarpal arthritis	T1 and T2 hypointense	Arthrodesis, PRC, arthroplasty

Treatment decisions are individualized and depend on symptom severity, patient activity level, and surgeon preference. Conservative therapy may suffice in early stages. Revascularization and joint-leveling osteotomies are options in intermediate stages. Salvage procedures such as proximal row carpectomy or wrist fusion are generally reserved for stage IV disease, as was done in our second and third cases [3,10,11].

## Conclusion

Kienböck’s disease is a rare but impactful wrist disorder that necessitates high clinical suspicion and appropriate imaging for diagnosis and staging. Although the Lichtman classification is useful for radiologic assessment, treatment must be individualized based on clinical presentation, functional needs, and surgical expertise. Our case series highlights the role of multimodal imaging and tailored management in achieving favorable outcomes.

## Patient consent

I, the author of the article « Kienbock's Disease: Case Report and Review of the Literature », declare that informed written consent was obtained from the patient for publication of the Case Report and all imaging studies in RADIOLOGY CASE REPORTS.
